# Key Features of Effective Yoga Interventions in Addition to Standard Medical Treatment for Rheumatoid Arthritis: A Systematic Review and Meta‐Analysis

**DOI:** 10.1002/acr2.70054

**Published:** 2025-05-10

**Authors:** Isha Biswas, Jaspreet Kaur, Fiona Pearce, Sarah Lewis, Kaushik Chattopadhyay

**Affiliations:** ^1^ Lifespan and Population Health, School of Medicine University of Nottingham Nottingham United Kingdom; ^2^ Division of Translational Medical Sciences, Centre of Biomolecular Sciences, School of Medicine University of Nottingham Nottingham United Kingdom; ^3^ Lifespan and Population Health, School of Medicine, University of Nottingham, Department of Rheumatology, Nottingham University Hospitals NHS Trust, and NIHR Nottingham Biomedical Research Centre Nottingham United Kingdom; ^4^ Lifespan and Population Health, School of Medicine, University of Nottingham and The Nottingham Centre for Evidence‐Based Healthcare Nottingham United Kingdom

## Abstract

**Objective:**

This systematic review aimed to synthesize the content, structure, and delivery characteristics of effective yoga interventions in addition to standard medical treatment for rheumatoid arthritis (RA).

**Methods:**

The Joanna Briggs Institute guidelines were followed. Seventeen databases were searched for randomized controlled trials (RCTs) assessing yoga's effectiveness in treating RA outcomes (disease activity score, pain, and function). Meta‐analyses and narrative synthesis were conducted.

**Results:**

Nine articles representing five RCTs were included and had low methodological quality scores. Yoga interventions, in addition to standard medical treatment, improved disease activity scores (standardized mean difference [SMD] −0.46, 95% confidence interval [CI] −0.73 to −0.18) and function (SMD −0.42, 95% CI −0.78 to −0.07) but did not effectively reduce pain (SMD −1.06, 95% CI −2.62 to 0.50) compared to standard medical treatment alone. All five RCTs found yoga's beneficial effects on one or more outcomes. All yoga interventions included center‐based (supervised, group) sessions, and two included additional home‐based (unsupervised, individual) sessions. All interventions incorporated 20 yogic poses (6 standing, 5 supine, 5 prone, and 4 seated), 7 breathing practices, and 4 meditation and relaxation practices. Two interventions offered RA‐specific yogic pose modifications. Center‐based sessions were delivered at least once weekly for 8 weeks’ median duration and around 68 minutes per session. Home‐based yoga was recommended thrice weekly for a 10‐week mean duration and 40 minutes per session.

**Conclusion:**

Yoga might be useful in addition to standard medical treatment for RA. Given previous studies’ methodological limitations, a high‐quality RCT should be conducted based on our synthesized key features of effective yoga interventions.

## INTRODUCTION

Rheumatoid arthritis (RA) is the most common autoimmune inflammatory arthritis, characterized by warm, swollen, painful, and stiff joints, and can result in poor joint function.^1^, ^2^ The physical symptoms and psychological distress in RA can negatively affect the individual's quality of life.^2^, ^3^, ^4^, ^5^, ^6^ In 2020, the number of RA‐prevalent cases worldwide was estimated to be 17.6 million, and the number of cases is projected to reach around 31.7 million^7^ by 2050. Given the significant impact of RA on individuals, there is a need for advancements in treatment alternatives to improve their condition and alleviate the burden on health care systems.^8^

The existing guidelines suggest a treat‐to‐target approach for RA treatment, which aims to achieve a target (ie, remission or reduced disease activity) through treatment options.^9^, ^10^, ^11^ Currently, this involves the administration of disease‐modifying antirheumatic drugs (DMARDs), for example, methotrexate, sulfasalazine, and hydroxychloroquine, and often glucocorticoids within three months of the onset of symptoms, followed by treatment escalation to biologic treatment and targeted treatments (eg, JAK inhibitors) after six months of treatment if remission has not been achieved.^10^, ^11^ Prolonged use of these medications may have adverse effects (eg, gastrointestinal distress) and economic burdens on individuals and the health care system.^12^, ^13^, ^14^ Recently, there has been a growing interest in complementary and alternative medicine for the treatment of inflammatory rheumatic conditions.^15^, ^16^, ^17^, ^18^ Further, the National Institute for Health and Care Excellence (NICE) guideline also recommends that complementary approaches in addition to conventional treatment could be beneficial in RA.^19^

Yoga, a popular complementary health approach, might be helpful for people with RA.^20^, ^21^, ^22^, ^23^ The ancient mind–body practice of yoga originated in the Indian subcontinent.^24^ It involves a holistic approach that includes major components such as yogic poses (asana), breathing practices (pranayama), and meditation (dhyana) and relaxation practices, along with the encouragement of a healthy lifestyle.^25^ Generally, yoga is easy, safe, and affordable; demands minimal supervision and equipment; and can be practiced indoors and outdoors.^24^, ^25^ The American College of Rheumatology (ACR) and the EULAR guidelines recommend using additional interventions (eg, mind–body exercises such as yoga) in conjunction with standard medications for RA treatment.^26^, ^27^, ^28^, ^29^

Some systematic reviews that included randomized and nonrandomized studies have reported beneficial effects of yoga interventions on one or more RA outcomes, such as lower disease activity scores, pain relief, and functional improvement.^30^, ^31^, ^32^, ^33^, ^34^, ^35^, ^36^ A recent systematic review and meta‐analysis that included both randomized and nonrandomized studies found a reduction in disease activity scores and improved physical function in those receiving a yoga intervention in addition to standard medical treatment compared to no additional intervention.^35^ Content, structure, and delivery characteristics are key features of a yoga intervention. Here, the content of yoga sessions means yogic poses (asana), breathing practices (pranayama), and meditation (dhyana) and relaxation practices. Structure refers to the duration and frequency of yoga sessions and the total intervention duration. Delivery characteristics of yoga sessions mean the intervention context, yoga instructors’ details, and strategies to enhance intervention uptake and adherence. Synthesizing these key features of effective interventions is crucial in developing a high‐quality yoga program aimed at improving RA outcomes. Although some of these reviews described characteristics of yoga interventions used in addition to standard medical treatment for RA, they did not synthesize their key features.^30^, ^31^, ^32^, ^33^, ^34^, ^35^, ^36^ Therefore, this systematic review and meta‐analysis aimed to meet this gap in the literature by synthesizing the content, structure, and delivery characteristics of effective yoga interventions in addition to standard medical treatment for RA. These findings could be used to develop a comprehensive evidence‐based yoga intervention for treating RA for wider implementation.

## MATERIALS AND METHODS

This systematic review adhered to the Joanna Briggs Institute (JBI) methodology for systematic reviews of effectiveness and the Preferred Reporting Items for Systematic Reviews and Meta‐Analyses (PRISMA) guidelines.^37^, ^38^ This review was conducted according to the *a priori* published protocol^39^ and was registered with PROSPERO (CRD42022320337). Two independent reviewers (IB and JK) conducted the study screening and selection, methodologic quality assessment, data extraction, and data synthesis and assessed the certainty of evidence. Any disagreement was resolved through discussion or involving a third reviewer (FP, SL, or KC).

### Inclusion criteria

#### Population

Studies conducted among adults (aged ≥18 years) diagnosed with RA were included. No restrictions were applied regarding the diagnostic criteria of RA, including diagnosis based on physical examination, blood tests, and/or joint scans (eg, diagnosis based on the ACR criteria^9^ or physician diagnosed).

#### Intervention

Studies that assessed yoga interventions in addition to standard medical treatment for RA including at least one of the major components of yoga; namely, asana (yogic poses), pranayama (breathing practices), and dhyana (meditation) and relaxation practices were included. There were no restrictions on the yoga style and its session length, frequency, duration, and delivery mode.

#### Comparator

Studies comparing yoga with no additional intervention (ie, participants received only standard medical treatment for RA), sham intervention, or nonpharmaceutical intervention (eg, educational intervention) were included. Studies with head‐to‐head comparisons of two or more yoga interventions (ie, different in terms of content, structure, or delivery characteristics) were excluded.

#### Outcome

Studies that assessed at least one of the main outcomes of RA (ie, disease activity score, pain, or function) were included.^19^, ^40^, ^41^ Studies reporting the Disease Activity Score in 28 joints (DAS28) and other composite scores used to measure disease activity were eligible.^9^, ^42^ Disease activity trajectory is commonly monitored using the DAS28 score, which includes objective measures assessed by the clinician (swollen joint counts and the markers of inflammation measured in blood tests [either erythrocyte sedimentation rate (ESR) or C‐reactive protein (CRP) level]) and the subjective patient‐reported measures (tender joint counts and global health as assessed by using a visual analog scale [VAS] of patient‐reported disease activity).^43^ The patient‐reported or subjective measures within the DAS28 composite score represent the patient global assessment and are more susceptible to individual‐level variation.^44^ Any scale used for assessing pain (eg, VAS) and function (eg, Arthritis Impact Measurement Scale) was eligible.^31^ Radiographic outcomes are not considered core outcomes and therefore were excluded.^40^

#### Study design

Considering the hierarchy of study designs, only randomized controlled trials (RCTs) were included. Nonrandomized pilot or feasibility trials were excluded.

### Data sources and search strategies

The following 13 databases were searched to find published studies from their inception dates to November 16, 2023: Medline (Ovid), Embase (Ovid), PsycInfo (Ovid), CINAHL (EBSCOHost), Cochrane Central Register of Controlled Trials (CENTRAL), Allied and Complementary Medicine (AMED) (Ovid), SPORTDiscus (EBSCOhost), Web of Science (Clarivate Analytics), Turning Research Into Practice (TRIP), AYUSH Research Portal, A Bibliography of Indian Medicine (ABIM), CAM‐QUEST, and Physiotherapy Evidence Database (PeDro). Unpublished studies were also searched from their inception dates to November 16, 2023, using OpenGrey (from 1997), EthOS (from 1925), ProQuest Dissertations and Theses (from 1980), and DART‐Europe e‐theses portal (from 1999). No language restrictions were applied. The search strategies were developed in consultation with a research librarian at the University of Nottingham (Supplementary Data S1) based on the search strategies in the following: “yoga” component based on a relevant systematic review,^45^ “RA” component based on the UK's NICE guideline on RA^46^ and a Cochrane systematic review on RA,^47^ and “RCT” component using predesigned search filters.^48^, ^49^, ^50^ The reference lists of all the included studies and relevant previous systematic reviews were screened for additional studies.

### Study screening and selection

Retrieved articles from the searches were uploaded to Endnote X9 (Clarivate Analytics),^51^ and after duplicate removal, the remaining citations were uploaded onto Rayyan (Qatar Computing Research Institute [Data Analytics])^52^ to facilitate the title and abstract screening process using the inclusion criteria. Studies identified as potentially eligible or those without an abstract had their full text retrieved. The full texts of the studies were assessed for eligibility, and those that did not meet the inclusion criteria were excluded, citing reasons.

### Methodologic quality assessment and data extraction

Methodologic quality assessment was conducted using the standardized JBI critical appraisal checklist for RCTs, assigning a score as met (yes), not met (no), unclear, or not applicable.^37^ Data was extracted using a predeveloped and pretested data extraction form. The authors extracted the end‐of‐intervention data (mean and SD) for all the outcomes (disease activity score, pain, and function).^40^, ^47^ When this time point was not reported, data from the time point closest to the end of the intervention were extracted. Yoga‐related adverse events, if reported, were extracted. In case of unclear data, the corresponding authors were contacted by email (twice) to obtain the relevant data.

### Data synthesis

Considering the errors in how authors analyze and report yoga interventions to be effective in studies (eg, conducting pre‐post analysis of outcomes within study arms but no comparative analysis between study arms), meta‐analyses were conducted for yoga in addition to standard medical treatment versus any comparator to determine the true effectiveness of each included yoga intervention for all the outcomes (disease activity score, pain, and function). Random‐effects meta‐analyses using Review Manager 5.4.1 (The Nordic Cochrane Centre, The Cochrane Collaboration) were conducted to identify effective yoga interventions.^53^ Because the included studies used different scales to report the outcomes, standardized mean differences (SMDs) with 95% confidence intervals (CIs) were estimated. It should be noted that calculating SMDs does not correct for differences in the direction of scales (ie, when some scales increase with disease severity, whereas others decrease).^54^ In such cases, the extracted mean values of scales in contrary directions were multiplied by (−1) to ensure consistency in the direction of scales, and SDs were used as reported.^54^ Subsequently, the content, structure, and delivery characteristics of the effective yoga interventions were narratively synthesized using tables and text.

### Assessing the certainty of the evidence

Certainty in the findings was assessed using the Grading of Recommendations Assessment, Development and Evaluation (GRADE) approach (for each outcome). The findings were initially ranked as high and downgraded to moderate, low, or very low if there was evidence of the following: risk of bias, inconsistency of results, indirectness of evidence, imprecision, and/or publication bias.^55^ Further details are described in Supplementary Data S2.

## RESULTS

### Study selection

**Figure 1:**
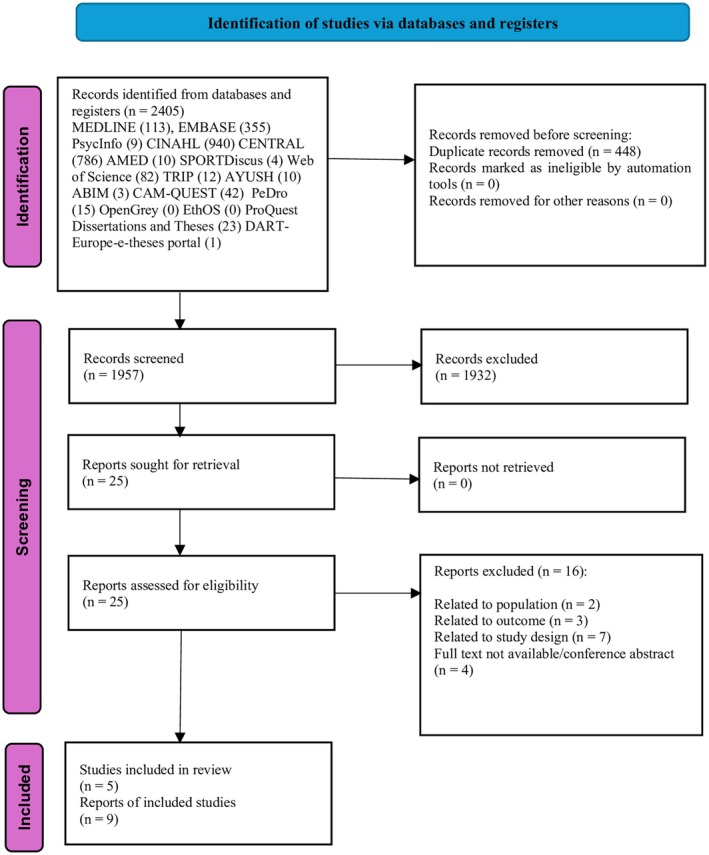
Preferred Reporting Items for Systematic Reviews and Meta‐Analyses flowchart for included studies from searches of databases and registers only.

A total of 2,405 records were identified through the literature search. After removal of duplicates and title and abstract screening, 25 articles were retrieved for full‐text screening. Nine articles were included in this systematic review, representing five studies (RCTs).^56^, ^57^, ^58^, ^59^, ^60^, ^61^, ^62^, ^63^, ^64^ Five articles described the same RCT, providing data on different outcomes and, therefore, were included as a single study in this review.^58^, ^59^, ^60^, ^61^, ^62^ The study selection process is detailed in the PRISMA flowchart (Figure 1). No additional articles were identified from citation searching. The list of articles excluded following the full‐text review is presented in Supplementary Data S3. During screening, no studies that used yoga as the sole intervention (ie, without standard medical treatment) or as part of other multimodal interventions were found.

### Description of the included studies

Five studies met the inclusion criteria, and 718 participants were included.^56^, ^57^, ^58^, ^59^, ^60^, ^61^, ^62^, ^63^, ^64^ Three studies were conducted in India^56^, ^58^, ^59^, ^60^, ^61^, ^62^, ^63^ (n = 635), and one each was conducted in New Zealand^57^ (n = 26) and Croatia^64^ (n = 57). The number of enrolled participants with RA ranged from 26 to 143. Studies recruited participants aged 18 to 75 years, with mean ages ranging from 35 years^56^ to 55 years.^64^ Of the 718 participants, most were women (n = 598; 83%). The mean duration of symptoms ranged from <3 years^63^ to 12 (SD 10) years.^57^ When mentioned (three of five studies), participants had no previous experience of yoga practice,^63^, ^64^ whereas 31% of participants had practiced yoga previously in another study.^57^ When reported (two of five studies), the mean disease severity was 2.60 (SD 0.85) for low disease activity^64^ and 4.89 (SD 0.81) for moderate disease activity,^58^, ^59^, ^60^, ^61^, ^62^ as per the DAS28‐ESR criteria. All studies reported prescribed medication use in yoga and control groups.^56^, ^57^, ^58^, ^59^, ^60^, ^61^, ^62^, ^63^, ^64^ When reported (two of five studies), the most commonly used medications were DMARDs among 77% of participants^64^ and 92% of participants,^57^ glucocorticoids among 30% of participants^64^ and 46% of participants,^57^ nonsteroidal anti‐inflammatory drugs among 9% of participants^62^ and 23% of participants,^55^ and biologic therapies among 18% of participants^64^ and 12% of participants.^57^ Some of the less frequently used medications by participants (reported in two of five studies) included methotrexate monotherapy^58^, ^59^, ^60^, ^61^, ^62^ (100%), methotrexate in combination with other DMARDs^58^, ^59^, ^60^, ^61^, ^62^ (33%), bone‐sparing therapies^57^ (31%), and analgesics^57^ (31%). The comparator groups varied from usual care (which included standard medication prescribed by a rheumatologist and following a daily routine with no involvement of an exercise regimen for the intervention duration^57^, ^58^, ^59^, ^60^, ^61^, ^62^), wait‐listed controls (ie, practicing the yoga intervention after 1.5 months^56^ and 3 months^63^ after the beginning of the intervention), to education involving a lecture by a rheumatologist on arthritis‐related topics facilitated by participant discussion.^64^ The overview of characteristics of the included studies and yoga intervention details of the included RCTs are reported in Table 1 and Supplementary Table S1, respectively.

**Table 1:** Characteristics of included studies*

Author and year	Country	Major inclusion criteria, including diagnosis	Population characteristics				Sample size, n	Intervention (yoga in addition to standard medical treatment)	Comparator (other interventions in addition to standard medical treatment)
			Age, mean ± SD, y	Sex, Female/Male	Duration of RA, mean ± SD, y	Any other therapy/medication for RA			
Singh et al,^56^ 2011	India	Ages 23–48 y; patients with RA; diagnosis: NR	Intervention: 35.07 ± 7.33; comparator: 34.65 ± 7.30	Both sexes, 56/24	Intervention: 11.52 ± 7.03; comparator: 12.15 ± 7.02	Usual medications for RA (NS)	80 (intervention: 40; comparator: 40)	Yoga (including cleaning practices and yogic diet)	Wait‐listed control
Ward et al,^57^ 2018	New Zealand	Age ≥18 y; average self‐reported pain over the previous month; ≥3 on a 10‐point NRS; average self‐reported sleep disturbance over the last month; diagnosis: by a physician using ACR/EULAR 2010 classification criteria^9^	All: 54.00 ± 11.00; intervention: 50.00 ± 12.00; comparator: 59.00 ± 8.00	Both sexes; intervention: 13/0; comparator: 12/1	All: 12.00 ± 10.00; intervention: 11.00 ± 10.00; comparator: 12.00 ± 11.00	DMARDs, NSAIDs, biologic therapies, bone‐sparing therapies, glucocorticoids, analgesics	26 (intervention: 13; comparator: 13)	Yoga	No additional intervention
Gautam et al,^58^ 2019	India	Age 18–60 y; routine medical treatment, including DMARDs ≥6 mo; diagnosis: ACR/EULAR 2010 classification criteria^9^	Intervention: 45.70 ± 1.60; comparator: 42.10 ± 1.70	Both sexes; intervention: 29/7; comparator: 27/9	Intervention: 6.32 ± 0.80; comparator: 5.61 ± 0.70	NR	72 (intervention: 36; comparator: 36)	Yoga	No additional intervention
Gautam et al,^59^ 2020			Intervention: 45.10 ± 8.70; comparator: 43.40 ± 9.30	Both sexes; intervention: 28/5; comparator: 25/8	NR	NR	66 (intervention: 33; comparator: 33)		
Gautam et al,^60^ 2021			Intervention: 45.60 ± 7.90; comparator: 44.50 ± 7.70	Both sexes; intervention: 29/6; comparator: 27/8	Intervention: 6.50 ± 4.6; comparator: 5.60 ± 4.2	NR	70 (intervention: 35; comparator: 35)		
Gautam et al,^61^ 2022			Intervention: 44.63 ± 11.90; comparator: 47.01 ± 12.00	Both sexes; intervention: 62/8; comparator: 56/14	Intervention: 6.09 ± 4.20; comparator: 6.10 ± 3.10	MTX monotherapy, MTX plus other DMARDs	140 (intervention: 70; comparator: 70)		
Gautam et al,^62^ 2023			Intervention: 46.0 ± 9.4; comparator: 41.8 ± 9.7	Both sexes; intervention: 26/6; comparator: 23/9	Intervention: 6.6 ± 4.8; comparator: 5.6 ± 4.4	MTX monotherapy, MTX plus other DMARDs	64 (intervention: 32; comparator: 32)		
Ganesan et al,^63^ 2020	India	Age 30–60 y; high disease activity (as per DAS28 criteria); diagnosis: ACR/EULAR 2010 classification criteria^9^	Intervention: 41.33; comparator: 42.59	Both sexes; intervention: 63/5; comparator: 68/7	<3	Standard medical treatment	143 (intervention: 68; comparator: 75)	Yoga	Wait‐listed control
Pukšić et al,^64^ 2021	Croatia	Age 18–75 y; low to moderate disease activity (DAS28‐CRP <5.1); stable standard pharmacotherapy for ≥3 mo; diagnosis: ACR/EULAR 2010 classification criteria^9^	Intervention: 52.90 ± 12.20; comparator: 57.90 ± 9.00	Both sexes; intervention: 30/0; comparator: 24/3	Intervention:7.40 ± 6.09; comparator: 8.70 ± 9.20	DMARDs, NSAIDs, biologic therapies, glucocorticoids	57 (intervention: 30; comparator: 27)	Yoga	Educational control (lecture by a rheumatologist on arthritis‐related topics^a^ plus participant discussion)

* Only yoga‐related interventions were mentioned under “Intervention,” and no intervention or any other active interventions were mentioned under “Comparator.” ACR, American College of Rheumatology; CRP, C‐reactive protein; DAS28, Disease Activity Score in 28 joints; DMARD, disease‐modifying antirheumatic drug; MTX, methotrexate; NR, not reported; NRS, numeric rating scale; NS, not specified; NSAID, nonsteroidal anti‐inflammatory drug; RA, rheumatoid arthritis.

^a^ For example, inflammatory process and joints, RA symptoms, comorbidities, therapy and treat‐to‐target approach, and exercise and self‐help.

### Methodologic quality of the included studies

Overall, the methodology was not adequately reported in the included studies, which resulted in low methodologic quality scores (total “yes” response percentage on the checklist ranging from 25% to 66%) (Supplementary Table S2). Some major issues included inadequate reporting of allocation concealment, imbalances between the characteristics of treatment groups at baseline, inadequate description of blinding of participants and outcome assessors, inadequate reporting of whether the study arms were treated identically other than the intervention of interest, no or insufficient analysis of the differences between study groups about the loss to follow‐up and reasons for incomplete follow‐up, inadequate reporting of the intent‐to‐treat analysis and its details, no or inadequate description of the number of outcome assessors or their training, missing information on statistical power analysis calculations and assumptions of statistical tests used, and errors in statistical analysis and reporting.

### Meta‐analysis to determine studies with effective yoga interventions and certainty of the evidence

**Figure 2:**
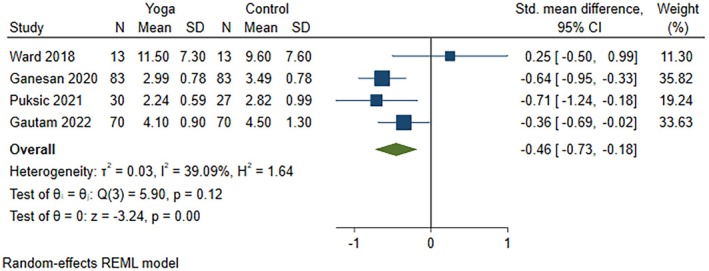
Forest plot for the outcome disease activity score, comparing yoga in addition to standard medical treatment versus the comparator group. Disease activity scores were reported in the studies by Gautam et al,^58^, ^59^, ^60^, ^61^, ^62^ but the values reported in the 2022 study by Gautam et al^61^ (largest sample size) were extracted. CI, confidence interval; N, sample size; REML, restricted maximum likelihood.

**Figure 3:**
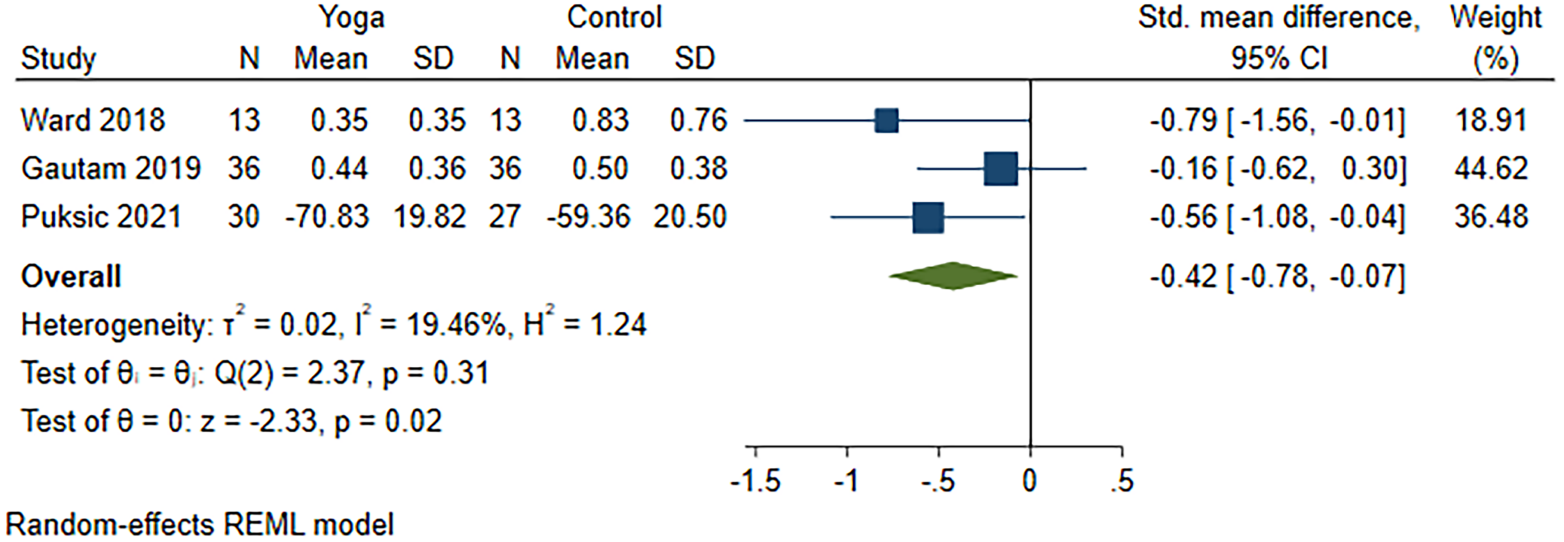
Forest plot for outcome the function, comparing yoga in addition to standard medical treatment versus the comparator group. Function scores were reported in the 2019 and 2021 studies by Gautam et al,^58^, ^60^ but the values reported in the 2019 study by Gautam et al^58^ were extracted because it had the largest sample size. CI, confidence interval; N, sample size; REML, restricted maximum likelihood.

**Figure 4:**
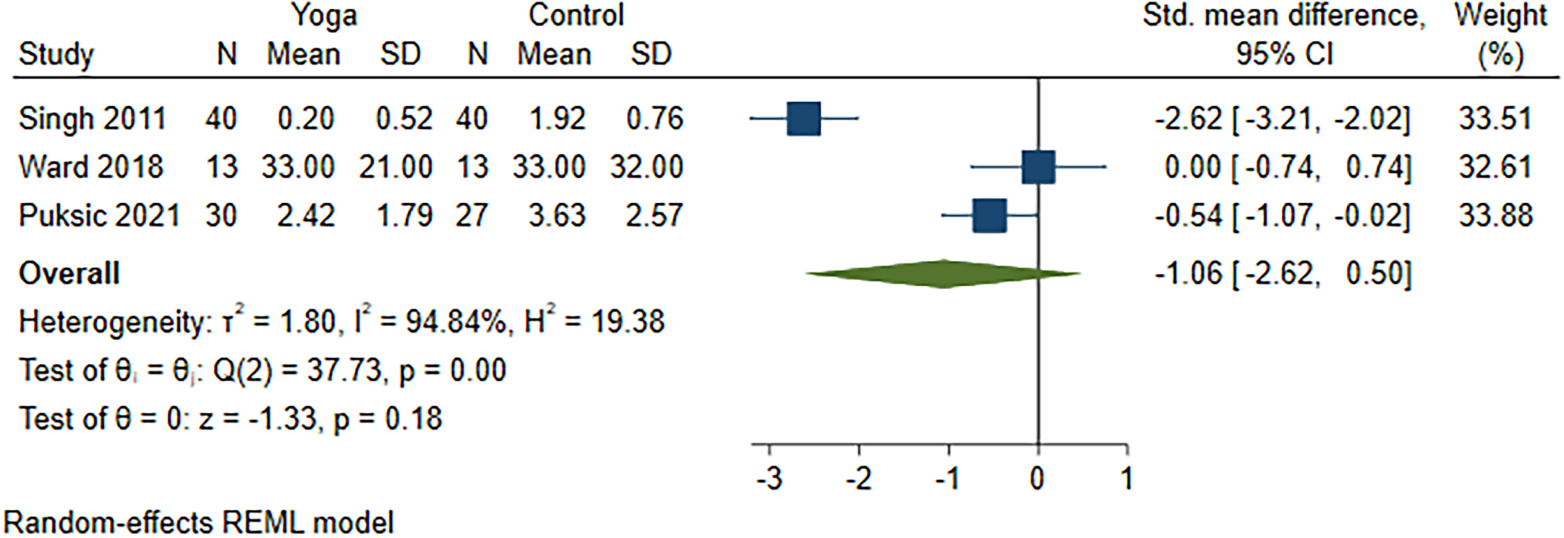
Forest plot for the outcome pain, comparing yoga in addition to standard medical treatment versus the comparator group. CI, confidence interval; N, sample size; REML, restricted maximum likelihood.

All five studies (nine articles) were included in the meta‐analysis to identify the individual effective interventions for each outcome: disease activity score (four studies), pain (three studies), and function (three studies). These studies compared yoga interventions in addition to standard medical treatment with the following comparators: (1) standard medical treatment including prescribed DMARDs, along with normal daily routine and not enrolling in any exercise regimen, aerobic activities, or yoga for the intervention duration^57^, ^58^, ^59^, ^60^, ^61^, ^62^; (2) wait‐listed control^56^, ^63^; and (3) education on arthritis‐related topics and discussion facilitated by a rheumatologist.^64^ All comparators in our included studies involved standard medications,^56^, ^57^, ^58^, ^59^, ^60^, ^61^, ^62^, ^63^, ^64^ specifically DMARDs,^57–62,64^ except that one comparator included an additional education component.^64^ Yoga interventions with medications reduced disease activity scores (four RCTs; SMD −0.46, 95% CI −0.73 to −0.18) (Figure 2) and improved function (three RCTs; SMD −0.42, 95% CI −0.78 to −0.07) (Figure 3) but were not effective in reducing pain (three RCTs; SMD −1.06, 95% CI −2.62 to 0.50) (Figure 4) as compared to comparators. Effective results for disease activity score, pain, and function were reported in three individual RCTs,^61^, ^63^, ^64^ two individual RCTs,^56^, ^64^ and two individual RCTs,^57^, ^64^ respectively. In other words, yoga interventions in all five included RCTs were effective on disease activity score, pain, and/or function.^56^, ^57^, ^58^, ^59^, ^60^, ^61^, ^62^, ^63^, ^64^ Extracted outcome data are reported in Supplementary Table S3, and an explanation of the specific scales used in each study is provided in Supplementary Table S4. Moderate certainty evidence for disease activity score and function and very low certainty evidence for pain were found using the GRADE approach (Supplementary Table S5).

### Content, structure, and delivery characteristics of effective yoga interventions for disease activity score, pain, and/or function

The yoga interventions of all five RCTs included center‐based (supervised, group) sessions,^56^, ^57^, ^58^, ^59^, ^60^, ^61^, ^62^, ^63^, ^64^ and two interventions had additional home‐based (unsupervised, individual) sessions.^57^, ^63^ The median duration was 8 weeks (interquartile range [IQR] 8–12 weeks), and each session was around 68 minutes (nearly 1 hour 10 minutes) (IQR 60–85 minutes), and these sessions were delivered in varied frequencies, ranging from once a week^57^ to daily.^56^ Home‐based yoga practice was recommended thrice weekly for a mean duration of 10 weeks and 40 minutes per session.^57^, ^63^

All five RCTs reported the major components of yoga used.^56^, ^57^, ^58^, ^59^, ^60^, ^61^, ^62^, ^63^, ^64^ The content of yoga interventions was heterogeneous and included 20 different yogic poses (asana; 6 standing, 5 supine, 5 prone, and 4 seated), 7 breathing practices (pranayama), and 4 meditation (dhyana) and relaxation practices. All included pranayama and dhyana and relaxation practices,^56^, ^57^, ^58^, ^59^, ^60^, ^61^, ^62^, ^63^, ^64^ and four also included asana.^56^, ^57^, ^58^, ^59^, ^60^, ^61^, ^62^, ^63^ None of these interventions mentioned the yoga style used. Interventions that included all the three major components of yoga reported the time allocated to each component.^56^, ^57^, ^58^, ^59^, ^60^, ^61^, ^62^, ^63^ The median time allocated to asana was around 29 minutes (IQR 17–39 minutes),^56^, ^57^, ^58^, ^59^, ^60^, ^61^, ^62^, ^63^ the median time allocated to pranayama was around 20 minutes (IQR 7–20 minutes),^56^, ^57^, ^58^, ^59^, ^60^, ^61^, ^62^, ^63^, ^64^ and the median time allocated to dhyana and relaxation practices was around 25 minutes (IQR 15–25 minutes).^56^, ^57^, ^58^, ^59^, ^60^, ^61^, ^62^, ^63^, ^64^ Some of the common practices were Pawanmuktasana (wind‐relieving pose),^56^, ^57^, ^58^, ^59^, ^60^, ^61^, ^62^, ^63^ Bhujangasana (cobra pose),^56^, ^58^, ^59^, ^60^, ^61^, ^62^, ^63^ Nadishodhana Pranayama (alternate nostril breathing),^56^, ^58^, ^59^, ^60^, ^61^, ^62^, ^63^, ^64^ Bhramari Pranayama (humming bee breathing),^56^, ^58^, ^59^, ^60^, ^61^, ^62^, ^63^ Nadanusandhana (A‐U‐M Kara chanting),^56^, ^58^, ^59^, ^60^, ^61^, ^62^, ^63^, ^64^ and Savasana (corpse pose).^56^, ^58^, ^59^, ^60^, ^61^, ^62^, ^63^ Supplementary Table S6 describes the various yoga practices effective for disease activity score, pain, and/or function.^65^, ^66^

When reported (one of five interventions), center‐based yoga sessions were delivered in a progressive manner (ie, new postures were introduced every two weeks and were upgraded from predominantly supine to standing posture).^57^ To accommodate participants’ functional limitations, supine postures were practiced on an electronic treatment table, which assisted the participants with self‐mobilization up and down from the floor, and alternative seated versions were taught for standing postures for those uncomfortable with weight‐bearing on their feet.^57^ Additionally, yoga postures were adapted (two of five interventions) using props such as exercise balls, blocks, belts, armless chairs, pillows, and blankets to accommodate participants’ needs (eg, to maintain correct postural alignment and avoid excess kneeling).^57^, ^64^ The use of CDs was reported to facilitate guided relaxation practices during home‐based yoga practice (one of two interventions).^57^

When reported (one of five interventions), strategies were used to monitor and improve adherence to yoga practice at the center, for example, by keeping track of yoga practice (self‐recorded by participants in yoga diaries shared with the yoga providers) and yoga providers’ remarks at each visit.^58^, ^59^, ^60^, ^61^, ^62^ Furthermore, strategies were also used to monitor and increase adherence to yoga practice at home, for example, by reminding and motivating the participants through phone calls,^63^ verbally sharing details of home practice with the yoga provider at the beginning of each session,^57^ and discussion of barriers and facilitators to home practice among the yoga participants.^57^ Participants were encouraged to incorporate yoga practice into their daily lives for the long term (two of five interventions).^58–62,64^

## DISCUSSION

Our systematic review identified that yoga, when delivered in addition to standard medical treatment for RA, might have beneficial effects on disease activity score, pain, and function, as also reported in previous systematic reviews.^35^, ^67^ However, the findings should be interpreted with caution because the certainty of the evidence assessed using the GRADE approach ranged from moderate to very low. Although reductions in disease activity and function scores were statistically significant, the clinical effectiveness, in terms of effect sizes, compared to published minimal clinically important differences, appears to be small to moderate.^68^ However, any improvement in disease activity and function scores can still be beneficial, especially when yoga is practiced in addition to standard medical treatment, and this review synthesized the key features of effective interventions, which are important for developing a high‐quality yoga intervention to improve RA outcomes. Supervised yoga practice in groups at a center was one of the most consistent characteristics of the yoga interventions. This might reflect the importance of creating a “community” for yoga practice and a space for personal interactions between the patients and the yoga provider during supervised yoga sessions. This could help them deal with negative emotions (eg, shame) and overcome social isolation, which are major concerns in RA.^69^ Participants’ adherence to yoga interventions is likely to play an important role in yoga's effectiveness on RA outcomes.^70^ Supervised programs are likely to have increased adherence to yoga sessions.^71^, ^72^ Another important finding of this review was that all the effective yoga interventions incorporated two major components: breathing practices and meditation and relaxation practices. It is possible that including both these components may work by stimulating the parasympathetic nervous system and relieving RA outcomes by reducing anxiety and stress.^73^, ^74^, ^75^

To the best of our knowledge, this is the first systematic review using a comprehensive search using a broad range of databases and a robust methodology to synthesize the key features of effective yoga interventions for treating RA and to assess the certainty of evidence using the GRADE approach. Only RCTs were included in the review, based on the hierarchy of study designs. Determination of the effectiveness of interventions was standardized across RCTs using meta‐analysis before finally synthesizing the detailed characteristics of effective yoga interventions. The systematic review and meta‐analysis had some limitations. Although we conducted a comprehensive search, only nine articles could be included. Although DAS28‐CRP values are lower than DAS28‐ESR values,^76^ possibly underestimating disease activity, and because there was a paucity of data for the conversion of DAS28‐CRP to DAS28‐ESR values in one included RCT, these were used interchangeably. This could be a limitation of our meta‐analysis. Furthermore, publication bias could not be assessed given the limited number of included RCTs. The included RCTs had limitations as well. Overall, the methodology was not adequately reported, resulting in their poor methodologic quality scores. Methodologic quality assessment is a subjective process, and because the systematic reviewers were strict in responding to the questions in the JBI checklist, it could have led to low‐quality scores. Given the statistical heterogeneity and small sample sizes, the quality of evidence for disease activity score and function was downgraded to moderate and the quality of evidence for pain was downgraded to very low using the GRADE approach.

Most included RCTs were conducted in India, which might be indicative of the origins of yoga from the Indian subcontinent. Evidence shows that yoga RCTs conducted in India have increased odds of reaching positive conclusions than those conducted elsewhere.^77^ This might limit the generalizability of our findings because they may not accurately represent the broader population of patients with RA, warranting future trials to be conducted in various countries for a wider geographic representation. Participants in the included RCTs were predominantly women. Although RA is more common in women, it also affects men. So, future trials must recruit more male participants with RA and undertake initiatives to overcome the potential barriers to yoga practice among men (eg, men‐only yoga classes to overcome gender‐based perceptions of yoga), ensuring their concerns are addressed.^78^, ^79^ Most RCTs did not specify yoga providers’ credentials; instead, they only described them as “qualified” or “experienced.” There was also no description of whether they were bespoke trained to deliver the specific intervention to the patients with RA. Yoga providers with a minimum level of training and expertise and specific training in the intervention protocol are crucial to the effectiveness of the intervention and in ensuring its fidelity and safety.^80^, ^81^, ^82^ Future RCTs should therefore ensure adequate reporting of these details. Yoga‐related adverse events were not reported in most RCTs, and this should be improved in future trials to indicate the safety of yoga. Only two RCTs reported encouragement of long‐term yoga practice. Because RA is a chronic condition, it will be important to explore the long‐term impact of yoga through follow‐up assessments at different periods. Future research should aim to develop a yoga intervention for RA using our synthesized findings and conduct a high‐quality RCT with a large enough sample size. The development work requires the involvement and engagement of patients with RA, rheumatologists, yoga experts and providers, rheumatology nurse specialists, occupational therapists, and physiotherapists. Input from them will help identify the effective components of the intervention using Delphi or other consensus techniques.^83^ If found to be effective and safe, its implementation and integration into health care systems will be important.^84^

Yoga might be useful in addition to standard medical treatment for RA. Given the methodologic limitations of previous studies, a high‐quality RCT based on our synthesized key features of effective yoga interventions should be conducted.

## AUTHOR CONTRIBUTIONS

All authors contributed to at least one of the following manuscript preparation roles: conceptualization AND/OR methodology, software, investigation, formal analysis, data curation, visualization, and validation AND drafting or reviewing/editing the final draft. As corresponding author, Miss Biswas confirms that all authors have provided the final approval of the version to be published and takes responsibility for the affirmations regarding article submission (eg, not under consideration by another journal), the integrity of the data presented, and the statements regarding compliance with institutional review board/Declaration of Helsinki requirements.

## Supporting information


**Disclosure form**.

**Supplementary Data S1:** Search strategies
**Supplementary Data S2:** Assessing the certainty of the evidence using GRADE approach
**Supplementary Data S3:** *Excluded studies with reasons for exclusion*
**Table S1:** Yoga intervention details (content, structure and delivery characteristics) of the included RCTs.
**Table S2:** Methodological assessment of the included studies.
**Table S3:** Extracted outcome data.
**Table S4:** Description of outcome measurement scales, including scoring, and interpretation.
**Table S5:** Summary of findings.
**Table S6:** Yoga practices used in effective interventions for disease activity score, pain, and/or function.
