# Supplementary material for: Key Features of Effective Yoga Interventions in Addition to Standard Medical Treatment for Rheumatoid Arthritis: A Systematic Review and Meta‐Analysis
Source: ACR Open Rheumatol. 2025 May 10;7(5):e70054. doi: 10.1002/acr2.70054 (PMC12064992; doi:10.1002/acr2.70054)
Supplement: Supplementary file 2 — Supplementary Data S1: Search strategies Supplementary Data S2: Assessing the certainty of the evidence using GRADE approach Supplementary Data S3: Excluded studies with reasons for exclusion Table S1: Yoga intervention details (content, structure and delivery characteristics) of the included RCTs. Table S2: Methodological assessment of the included studies. Table S3: Extracted outcome data. Table S4: Description of outcome measurement scales, including scoring, and interpretation. Table S5: Summary of findings. Table S6: Yoga practices used in effective interventions for disease activity score, pain, and/or function. [file ACR2-7-e70054-s002.docx]

**Supplementary Data S1:** **Search strategies**

**MEDLINE (Ovid) <1946 to November 16, 2023 >: 113 records**

1 exp Mind-Body Therapies/

2 mind body therap*.mp.

3 meditat*.mp.

4 (yoga* or yogi*).mp.

5 (Asana* or Pranayam* or Dhyan* or Ashtanga or Bikram or Hatha or Iyengar or Kripalu or Kundalini or Vinyasa or Raja or Radja or Bhakti or Jnana or Kriya* or Karma or Yama or Niyama or Pratyahara or Dharana or Samadhi or Bandha or Mudra* or Chanda or Sivananda).mp.

6 1 or 2 or 3 or 4 or 5

7 exp Arthritis, Rheumatoid/

8 ((rheumatoid or reumatoid or revmatoid or rheumatic or reumatic or revmatic or rheumat* or reumat* or revmarthrit*) adj3 (arthrit* or artrit* or diseas* or condition* or nodule*)).mp.

9 (Sjogren* adj2 syndrome).mp.

10 (sicca adj2 syndrome).mp.

11 still* disease.mp.

12 bechterew* disease.mp.

13 (caplan* adj2 syndrome).mp.

14 (felty* adj2 syndrome).mp.

15 (rheumatoid adj2 factor*).mp.

16 ((inflammatory or idiopathic) adj2 (arthritis or polyarthritis)).mp.

17 8 or 9 or 10 or 11 or 12 or 13 or 14 or 15 or 16

18 randomized controlled trial.pt.

19 controlled clinical trial.pt.

20 randomized.ab.

21 placebo.ab.

22 drug therapy.fs.

23 randomly.ab.

24 trial.ab.

25 groups.ab.

26 18 or 19 or 20 or 21 or 22 or 23 or 24 or 25

27 exp animals/ not humans.sh.

28 26 not 27

29 6 and 17 and 28

**EMBASE (Ovid) <1974 to November 16, 2023 >: 355 records**

1 exp alternative medicine/

2 exp yoga/

3 exp meditation/

4 Mind body therap*.mp.

5 (Yoga* or yogi*).mp.

6 Meditat*.mp.

7 (Asana* or Pranayam* or Dhyan* or Ashtanga or Bikram or Hatha or Iyengar or Kripalu

or Kundalini or Vinyasa or Raja or Radja or Bhakti or Jnana or Kriya* or Karma or Yama or

Niyama or Pratyahara or Dharana or Samadhi or Bandha or Mudra* or Chanda or

Sivananda).mp.

8 1 or 2 or 3 or 4 or 5 or 6 or 7

9 exp rheumatoid arthritis/

10 ((rheumatoid or reumatoid or revmatoid or rheumatic or reumatic or revmatic or rheumat$

or reumat$ or revmarthrit$) adj3 (arthrit$ or artrit$ or diseas$ or condition$ or nodule$)).mp.

11 (sjogren$ adj2 syndrome).mp.

12 (sicca adj2 syndrome).mp.

13 still$ disease.mp.

14 bechterew$ disease.mp.

15 (caplan* adj2 syndrome).mp.

16 (felty* adj2 syndrome).mp.

17 (rheumatoid adj2 factor*).mp.

18 ((inflammatory or idiopathic) adj2 (arthritis or polyarthritis)).mp.

19 9 or 10 or 11 or 12 or 13 or 14 or 15 or 16 or 17 or 18

20 Randomized controlled trial/

21 Controlled clinical trial/

22 random*.ti,ab.

23 randomization/

24 intermethod comparison/

25 placebo.ti,ab.

26 (compare or compared or comparison).ti.

27 ((evaluated or evaluate or evaluating or assessed or assess) and (compare or compared or

comparing or comparison)).ab.

28 (open adj label).ti,ab.

29 ((double or single or doubly or singly) adj (blind or blinded or blindly)).ti,ab.

30 double blind procedure/

31 parallel group*1.ti,ab.

32 (crossover or cross over).ti,ab.

33 ((assign* or match or matched or allocation) adj5 (alternate or group*1 or intervention*1

or patient*1 or subject*1 or participant*1)).ti,ab.

34 (assigned or allocated).ti,ab.

35 (controlled adj7 (study or design or trial)).ti,ab.

36 (volunteer or volunteers).ti,ab.

37 human experiment/

38 trial.ti.

39 20 or 21 or 22 or 23 or 24 or 25 or 26 or 27 or 28 or 29 or 30 or 31 or 32 or 33 or 34 or 35

or 36 or 37 or 38

40 (random* adj sampl* adj7 (cross section* or questionnaire*1 or survey* or

database*1)).ti,ab. not (comparative study/ or controlled study/ or randomi?ed controlled.ti,ab.

or randomly assigned.ti,ab.)

41 Cross-sectional study/ not (randomized controlled trial/ or controlled clinical study/ or

controlled study/ or randomi?ed controlled.ti,ab. or control group*1.ti,ab.)

42 (((case adj control*) and random*) not randomi?ed controlled).ti,ab.

43 (Systematic review not (trial or study)).ti.

44 (nonrandom* not random*).ti,ab.

45 Random field*.ti,ab.

46 (random cluster adj3 sampl*).ti,ab.

47 (review.ab. and review.pt.) not trial.ti.

48 we searched.ab. and (review.ti. or review.pt.)

49 update review.ab.

50 (databases adj4 searched).ab.

51 (rat or rats or mouse or mice or swine or porcine or murine or sheep or lambs or pigs or

piglets or rabbit or rabbits or cat or cats or dog or dogs or cattle or bovine or monkey or

monkeys or trout or marmoset$1).ti. and animal experiment/

52 Animal experiment/ not (human experiment/ or human/)

53 40 or 41 or 42 or 43 or 44 or 45 or 46 or 47 or 48 or 49 or 50 or 51 or 52

54 39 not 53

55 8 and 19 and 54

**PsycInfo (OVID) <1806 to November 16, 2023>: 9 records**

1 exp Mind Body Therapy/

2 Mind-body therap*.mp.

3 exp Yoga/

4 (Yoga* or yogi*).mp.

5 exp Meditation/

6 Meditat*.mp.

7 (Asana* or Pranayam* or Dhyan* or Ashtanga or Bikram or Hatha or Iyengar or Kripalu

or Kundalini or Vinyasa or Raja or Radja or Bhakti or Jnana or Kriya* or Karma or Yama or

Niyama or Pratyahara or Dharana or Samadhi or Bandha or Mudra* or Chanda or

Sivananda).mp.

8 1 or 2 or 3 or 4 or 5 or 6 or 7

9 exp Rheumatoid Arthritis/

10 ((rheumatoid or reumatoid or revmatoid or rheumatic or reumatic or revmatic or rheumat*

or reumat* or revmarthrit*) adj3 (arthrit* or artrit* or diseas* or condition* or nodule*)).mp.

11 (Sjogren* adj2 syndrome).mp.

12 (sicca adj2 syndrome).mp.

13 still* disease.mp.

14 bechterew* disease.mp.

15 (caplan* adj2 syndrome).mp.

16 (felty* adj2 syndrome).mp.

17 (rheumatoid adj2 factor*).mp.

18 ((inflammatory or idiopathic) adj2 (arthritis or polyarthritis)).mp.

19 9 or 10 or 11 or 12 or 13 or 14 or 15 or 16 or 17 or 18

20 (Randomized Controlled Trial or Controlled Clinical Trial or Pragmatic Clinical Trial or

Equivalence Trial or Clinical Trial, Phase III).pt.

21 Randomized Controlled Trial/

22 exp Randomized Controlled Trials/

23 "Randomized Controlled Trial (topic)"/

24 Controlled Clinical Trial/

25 Controlled Clinical Trials/

26 exp Clinical Trials/

27 "Controlled Clinical Trial (topic)"/

28 Randomization/

29 Random Allocation/

30 Double-Blind Method/

31 Double Blind Procedure/

32 Double-Blind Studies/

33 Single-Blind Method/

34 Single Blind Procedure/

35 Single-Blind Studies/

36 Placebos/

37 Placebo/

38 Control Groups/

39 Control Group/

40 (random* or sham or placebo*).ti,ab,hw.

41 ((singl* or doubl*) adj (blind* or dumm* or mask*)).ti,ab,hw.

42 ((tripl* or trebl*) adj (blind* or dumm* or mask*)).ti,ab,hw.

43 (control* adj3 (study or studies or trial* or group*)).ti,ab.

44 (Nonrandom* or non random* or non-random* or quasi-random* or

quasirandom*).ti,ab,hw.

45 allocated.ti,ab,hw.

46 ((open label or open-label) adj5 (study or studies or trial*)).ti,ab,hw.

47 ((equivalence or superiority or non-inferiority or noninferiority) adj3 (study or studies or

trial*)).ti,ab,hw.

48 (pragmatic study or pragmatic studies).ti,ab,hw.

49 ((pragmatic or practical) adj3 trial*).ti,ab,hw.

50 ((quasiexperimental or quasi-experimental) adj3 (study or studies or trial*)).ti,ab,hw.

51 (phase adj3 (III or "3") adj3 (study or studies or trial*)).ti,hw.

52 20 or 21 or 22 or 23 or 24 or 25 or 26 or 27 or 28 or 29 or 30 or 31 or 32 or 33 or 34 or 35

or 36 or 37 or 38 or 39 or 40 or 41 or 42 or 43 or 44 or 45 or 46 or 47 or 48 or 49 or 50 or 51

53 8 and 19 and 52

**CINAHL (EBSCOHost) <1994 to November 16, 2023 >: 940 records**

S1 (MH "Mind Body Techniques+")

S2 TX Yoga* or yogi*

S3 TX "Mind body therap*"

S4 TX Meditat*

S5 TX Asana* or Pranayam* or Dhyan* or Ashtanga or Bikram or Hatha or Iyengar or Kripalu

or Kundalini or Vinyasa or Raja or Radja or Bhakti or Jnana or Kriya* or Karma or Yama or

Niyama or Pratyahara or Dharana or Samadhi or Bandha or Mudra* or Chanda or Sivananda

S6 S1 OR S2 OR S3 OR S5

S7 (MH "Arthritis, Rheumatoid+")

S8 TX Rheumatoid arthritis

S9 TX Sjogren* syndrome

S10 TX Sicca syndrome

S11 TX Still* disease

S12 TX Bechterew disease

S13 TX Caplan syndrome

S14 TX Felty* syndrome

S15 TX rheumatoid N2 factor*

S16 TX (inflammatory OR idiopathic) N2 (arthritis or polyarthritis)

S17 S7 OR S8 OR S9 OR S10 OR S11 OR S12 OR S13 OR S14 OR S15 OR S16

S18 TX randomized controlled trials

S19 MH double-blind studies

S20 MH single-blind studies

S21 MH random assignment

S22 MH pretest-posttest design

S23 MH cluster sample

S24 TI (randomised OR randomized)

S25 AB (random*)

S26 TI (trial)

S27 (MH (sample size) AND AB (assigned OR allocated OR control))

S28 MH (placebos)

S29 PT (randomized controlled trial)

S30 AB (control W5 group)

S31 MH (crossover design)

S32 MH (comparative studies)

S33 AB (cluster W3 RCT))

S34 S18 OR S19 OR S20 OR S21 OR S22 OR S23 OR S24 OR S25 OR S26 OR S27 OR S28

OR S29 OR S30 OR S31 OR S32 OR S33

S35 ((MH animals+ OR MH animal studies OR TI animal model*) NOT MH human)

S36 S34 NOT S35

S37 S6 AND S17 AND S36

**Cochrane Central Register of Controlled Trials (CENTRAL) <1996 to November 16,**

**2023>: 786 records**

#1 MeSH descriptor: [Mind-Body Therapies] explode all trees

#2 Meditation (Word variations have been searched)

#3 yoga* (Word variations have been searched)

#4 Mind body therapies (Word variations have been searched)

#5 (asana* or pranayam* or dhyan* or meditat* or ashtanga or bikram or hatha or iyengar or

kripalu or kundalini or vinyasa or raja or radja or bhakti or jnana or kriya* or karma or yama

or niyama or pratyahara or dharana or samadhi or bandha or mudra* or chanda or sivananda)

#6 #1 or #2 or #3 or #4 or #5

#7 MeSH descriptor: [Arthritis, Rheumatoid] explode all trees

#8 (Sjogren* adj2 syndrome).mp. (Word variations have been searched)

#9 (Sicca adj2 syndrome).mp. (Word variations have been searched)

#10 Still* disease.mp. (Word variations have been searched)

#11 Bechterew* disease.mp. (Word variations have been searched)

#12 (Caplan* adj2 syndrome).mp. (Word variations have been searched)

#13 (Felty* adj2 syndrome).mp. (Word variations have been searched)

#14 (rheumatoid adj2 factor*).mp. (Word variations have been searched)

#15 ((inflammatory or idiopathic) adj2 (arthritis or polyarthritis)).mp. (Word variations have

been searched)

#16 #7 OR #8 OR #9 OR #10 OR #11 OR #12 OR #13 OR #14 OR #15

#17 MeSH descriptor: [Randomized Controlled Trial] explode all trees

#18 MeSH descriptor: [Controlled Clinical Trial] explode all trees

#19 placebo* (Word variations have been searched)

#20 ("randomised clinical trial") (Word variations have been searched)

#21 (trial*) (Word variations have been searched)

#22 MeSH descriptor: [Randomized Controlled Trials as Topic] explode all trees

#23 #17 OR #18 OR #19 OR #20 OR #21 OR #22

#24 #6 AND #16 AND #23

**Allied and Complementary Medicine (AMED) (Ovid) <1985 to November 16, 2023>: 10**

**records**

1 exp Mind body medicine/

2 exp Yoga/

3 exp Meditation/

4 Mind body medicine*.mp.

5 (Yoga* or yogi*).mp.

6 Meditat*.mp.

7 (Asana* or Pranayam* or Dhyan* or Ashtanga or Bikram or Hatha or Iyengar or Kripalu

or Kundalini or Vinyasa or Raja or Radja or Bhakti or Jnana or Kriya* or Karma or Yama or

Niyama or Pratyahara or Dharana or Samadhi or Bandha or Mudra* or Chanda or

Sivananda).mp.

8 1 or 2 or 3 or 4 or 5 or 6 or 7

9 exp Arthritis rheumatoid/

10 ((rheumatoid or reumatoid or revmatoid or rheumatic or reumatic or revmatic or rheumat*

or reumat* or revmarthrit*) adj3 (arthrit* or artrit* or diseas* or condition* or nodule*)).mp.

11 (Sjogren* adj2 syndrome).mp.

12 (sicca adj2 syndrome).mp.

13 still* disease.mp.

14 bechterew* disease.mp.

15 (caplan* adj2 syndrome).mp.

16 (felty* adj2 syndrome).mp.

17 (rheumatoid adj2 factor*).mp.

18 ((inflammatory or idiopathic) adj2 (arthritis or polyarthritis)).mp.

19 9 or 10 or 11 or 12 or 13 or 14 or 15 or 16 or 17 or 18

20 (random* or factorial* or placebo* or assign* or allocat* or crossover*).tw.

21 (cross adj over*).tw.

22 (trial* and (control* or comparative)).tw.

23 ((blind* or mask*) and (single or double or triple or treble)).tw.

24 (treatment adj arm*).tw.

25 (control* adj group*).tw.

26 (phase adj (III or three)).tw.

27 (versus or vs).tw.

28 rct.tw.

29 RANDOM ALLOCATION/

30 DOUBLE BLIND METHOD/

31 placebos/

32 randomized controlled trials/

33 20 or 21 or 22 or 23 or 24 or 25 or 26 or 27 or 28 or 29 or 30 or 31 or 32

34 8 and 19 and 33

**SPORTDiscus (EBSCOHost) <2004 to November 16, 2023>: 4 records**

S1 SU Mind body therapy

S2 SU Yoga

S3 SU Meditation

S4 TX Mind body therap*

S5 TX Yoga* or yogi*

S6 TX Meditat*

S7 TX Asana* or Pranayam* or Dhyan* or Ashtanga or Bikram or Hatha or Iyengar or Kripalu

or Kundalini or Vinyasa or Raja or Radja or Bhakti or Jnana or Kriya* or Karma or Yama or

Niyama or Pratyahara or Dharana or Samadhi or Bandha or Mudra* or Chanda or Sivananda

S8 S1 OR S2 OR S3 OR S4 OR S5 OR S6 OR S7

S9 SU rheumatoid arthritis

S10 TX Rheumatoid arthritis

S11 TX Sjogren* syndrome

S12 TX Sicca syndrome

S13 TX Still* disease

S14 TX Bechterew disease

S15 TX Caplan syndrome

S16 TX Felty* syndrome

S17 TX rheumatoid N2 factor*

S18 TX (inflammatory OR idiopathic) N2 (arthritis or polyarthritis)

S19 S9 OR S10 OR S11 OR S12 OR S13 OR S14 OR S15 OR S16 OR S17 OR S18

S20 TX Randomized controlled trials

S21 SU Randomized controlled trials

S22 TX Double-blind studies

S23 TX Single-blind studies

S24 TX Random assignment

S25 TX Pretest-posttest design

S26 TX Cluster sample

S27 TX Placebos

S28 TX randomised or randomized

S29 TX random*

S30 TX trial*

S31 S21 OR S22 OR S23 OR S24 OR S25 OR S26 OR S27 OR S28 OR S29 OR S30 OR S30

S32 S8 AND S19 AND S31

**Web of Science <1998 to November 16, 2023>: 82 records**

#1 ALL=(yoga* OR “mind body therap*” OR meditation OR yogi* OR asana* OR pranayam*

OR dhyan* OR meditat* OR ashtanga OR bikram OR hatha OR iyengar OR kripalu OR

kundalini OR vinyasa OR raja OR radja OR bhakti OR jnana OR kriya* OR karma OR yama

OR niyama OR pratyahara OR dharana OR samadhi OR bandha OR mudra* OR chanda OR

sivananda)

#2 ALL=("Rheumatoid arthritis" or "sjogren* syndrome" or "sicca syndrome" or "still*

disease" or "bechterew* disease" or "caplan* syndrome" or "felty* syndrome" or rheumatoid

factor or "inflammatory arthritis" or "idiopathic arthritis" or "inflammatory polyarthritis")

#3 ALL=((“randomized controlled trial” OR “controlled clinical trial” OR “clinical trial” OR

“clinical trials" OR placebo* OR "random allocation" OR "double-blind method" OR "single-

blind method" OR "cross-over studies"))

#4 (ALL=((“randomized controlled trial” OR “controlled clinical trial” OR “clinical trial” OR

“clinical trials" OR placebo* OR "random allocation" OR "double-blind method" OR "single-

blind method" OR "cross-over studies"))) AND ALL=((randomised OR randomized OR

randomisation OR randomisation OR placebo* OR (random* AND (allocat* OR assign*)) OR

(blind* AND (single OR double OR treble OR triple))))

#5 #3 OR #4

#6 #1 AND #2 AND #5

**Turning Research Into Practice (TRIP); < 2014 to November 16, 2023>: 12 records**

**(RCTs)**

(yoga* OR "mind body therapies" OR yogi* OR asana* OR pranayam* OR dhyan* OR

meditation OR meditate OR ashtanga OR bikram OR hatha OR iyengar OR kripalu OR

kundalini OR vinyasa OR raja OR radja OR bhakti OR jnana OR kriya* OR karma OR yama

OR niyama OR pratyahara OR dharana OR samadhi OR bandha OR mudra* OR chanda OR

sivananda) AND ("rheumatoid arthritis" OR "sjogren* syndrome" OR "sicca syndrome"

OR "still* disease" OR "bechterew* disease" OR "caplan* syndrome" OR "felty* syndrome"

OR "rheumatoid factor" or inflammatory arthritis OR idiopathic arthritis OR inflammatory

polyarthritis) AND (randomised controlled trial OR randomized controlled trial OR controlled

clinical trial* OR clinical trial* OR placebo* OR random*)

**AYUSH Research Portal (Ministry of AYUSH, Government of India) <Date of search:**

**November 16, 2023: 10 records**

Medical system: yoga and naturopathy

Category: preclinical research, clinical research (Evidence grade - A, B, C) and fundamental

research

Body system: musculoskeletal

Disease (English): Rheumatoid/seropositive arthritis (ICPC-L88, ICD-M05, M06, M08, M45)

EVIDENCE GRADE – A:4    EVIDENCE GRADE – B:6     EVIDENCE GRADE – C:0

**A Bibliography of Indian Medicine (ABIM)<Date of search: November 16, 2023>: 3**

**records**

Search terms used: yoga and rheumatoid arthritis

**Complementary and alternative medicine (CAM-QUEST) < Date of search: November**

**16, 2023>: 42 records**

Searched for the following:

Disease pattern: pain (1155 results) to disease: chronic pain (209 results) to therapy: mind body medicine (75

results) to study design: randomized trial (33 results)

Searched for the following:

Therapy: Mind-body medicine (3708 results) to disease pattern: Musculoskeletal-/Connective tissue system (388

results) to disease: Arthritis (24 results) to study design: Randomized trial (13 results)

**Physiotherapy Evidence Database (PeDro) <from 1999 to November 16, 2023>: 15**

**records**

Searched terms used: yoga and rheumatoid arthritis

**OpenGrey (Data Archiving and Network Services) <from 1997 to November 16, 2023>:**

**0 records**

Search terms used: Yoga and rheumatoid arthritis

**EthOS <from 2009 to August 26, 2022>: 0 records**

Search terms used: yoga and rheumatoid arthritis

**ProQuest Dissertations and Theses (via Web of Science) <from 1902 to November 16,**

**2023>: 23 records**

#1 TS=((yoga* OR mind body therap* OR yogi* OR asana* OR pranayam* OR dhyan* OR

meditat* OR ashtanga OR bikram OR hatha OR iyengar OR kripalu OR kundalini OR vinyasa

OR raja OR radja OR bhakti OR jnana OR kriya* OR karma OR yama OR niyama OR

pratyahara OR dharana OR samadhi OR bandha OR mudra* OR chanda OR sivananda))

#2 TS=(("rheumatoid arthritis" OR "sjogren* syndrome" OR "sicca syndrome" OR "still*

disease" OR "bechterew* disease" OR "caplan* syndrome" OR "felty* syndrome" OR

"rheumatoid factor" or inflammatory arthritis OR idiopathic arthritis OR inflammatory

polyarthritis))

#3 TS=((randomised controlled trial OR randomized controlled trial OR controlled clinical

trial* OR clinical trial* OR placebo* OR random* or trial*))

#4 #1 AND #2 AND #3

**DART-Europe-e-theses portal <from 2005 to November 16, 2023>: 1 record**

Search terms used: yoga and rheumatoid arthritis

**Supplementary Data S2: Assessing the certainty of the evidence using GRADE approach**

In the risk of bias domain, the following were considered for downgrading: lack of allocation

concealment (no to Q2 in the JBI critical appraisal checklist for RCTs), lack of blinding (no to

Q4,5,7), attrition bias (no to Q11), and if there was selective outcome reporting. If an issue out

of these four was present in most studies (i.e., >50%), it was downgraded by one level. If more

than one issue was present, it was downgraded by two levels. In the inconsistency of results

domain, if the statistical heterogeneity (i.e., I ^2^ statistic) was 75%–89%, it was downgraded by

one level. If the I ^2^ statistic was ≥90%, it was downgraded by two levels. In the imprecision

domain, if the total sample size was 100 to <400, it was downgraded by one level. If the total

sample size was <100, it was downgraded by two levels. If the total sample size was ≥400 with

wide CI and not earlier downgraded for inconsistency, it was downgraded by one level. There

was no indirectness of evidence and publication bias could not be detected (<10 studies).

**Supplementary Data S3: Excluded studies with reasons for exclusion**

**Full-text studies excluded (n = 16)**

**Related to population (ineligible age group) (n = 2)**

Evans S, Moieni M, Lung K, et al. Impact of Iyengar yoga on quality of life in young women

with rheumatoid arthritis. Clin J Pain 2013;29:988-97.

Middleton KR, Ward MM, Moonaz SH, et al. Feasibility and assessment of outcome measures

for yoga as self-care for minorities with arthritis: a pilot study. Pilot Feasibility Stud

2018:10.1186/s40814-018-0248-x.

**Related to outcome (ineligible outcomes e.g., inflammation markers) (n = 3)**

**Dubey V. Efficacy of yoga therapy in rheumatoid arthritis patients with normal values on**

**measures of inflammation.** Int J Rheum Dis **2016;19:21.**

Dash M, Telles S. Improvement in hand grip strength in normal volunteers and rheumatoid

arthritis patients following yoga training. Indian J Physiol Pharmacol 2001;45:355-60.

Moonaz SH, Bingham CO, Wissow L, et al. Yoga in sedentary adults with arthritis: effects

of a randomized controlled pragmatic trial. J Rheumatol 2015;42:1194-202.

**Related to study design (e.g., pilot study (unclear if randomised), quasi-RCT, study**

**protocol) (n = 7)**

Evans S, Moieni M, Taub R, et al. Iyengar yoga for young adults with rheumatoid arthritis:

results from a mixed-methods pilot study. J Pain Symptom Manage 2010;39:904-13.

Evans S, Cousins L, Tsao JC, et al. A randomized controlled trial examining Iyengar yoga for

young adults with rheumatoid arthritis: a study protocol. Trials 2011;12: [10.1186/1745621512-19](https://doi.org/10.1186/1745-6215-12-19).

Ward L, Stebbings S, Athens J, et al. Yoga for pain and sleep quality in rheumatoid arthritis:

study protocol for a pilot randomized controlled trial. Phys Ther Rev 2014;19:266-76.

Bosch PR, Traustadóttir T, Howard P, et al. Functional and physiological effects of yoga

in women with rheumatoid arthritis: a pilot study. Altern Ther Health Med 2009;15:24-31.

Badsha H, Chhabra V, Leibman C, et al. The benefits of yoga for rheumatoid arthritis: results

of a preliminary, structured 8-week program. Rheumatol Int 2009;29:1417-21.

Greenfield RH. Flexible? Yoga and rheumatoid arthritis. Integrative Medicine Alert

2009;12:29-30.

Zochling J. Update on complementary and alternative medicines for arthritis. Medicine Today

2009;10:71-6.

**Full text not available/conference abstract (n = 4)**

Puksic S, Mitrovic J, Culo MI, et al Yoga leads to sustained improvement in fatigue and mood in rheumatoid arthritis: preliminary results of a randomized controlled trial. Ann Rheum Dis 2020;79:528.

Mahidashtizad S, Salajegheh A. Effects of a yoga program on pain relief, reducing anxiety and extended range of motion of knee in rheumatoid arthritis patients. Annals of the Rheumatic Diseases 2013;71:756.

Haslock I, Monro R, Nagarathna R, et al. Measuring the effects of yoga in rheumatoid arthritis. Rheumatol 1994;33:787–90.

Gautam S, Chaurasia P, Rana D, et al. Yoga reduces sperm oxidative DNA damage and improves fertility potential in infertile men with rheumatoid arthritis on disease-modifying antirheumatic drugs. 45^th^ Annual Meeting of American Society of Andrology (ASA); 2020 Apr; virtual.

Table S1: Yoga intervention details (content, structure and delivery characteristics) of the included RCTs.

| **Author and year** | **Intervention development** | **Intervention duration** | **Yoga sessions: content** | | | **Yoga sessions: structure (duration and frequency)** | **Yoga sessions: delivery characteristics (context, instructor, and uptake and adherence)** | **Extra features** |
| --- | --- | --- | --- | --- | --- | --- | --- | --- |
|  | | | **Asana** | **Pranayama** | **Dhyana and relaxation** |  |  |  |
| Singh, 2011^56^ | Based on ancient yoga texts to improve/alleviate pain, inflammation, stiffness, PR, BP, LC, CRP, and UA. | 7 weeks | **Asana - 40 mins**   - Anti-rheumatic series (Pawanmuktasana/   joint loosening series) for 1st week   - Marjari asana (cat pose) - Vakranasana (seated spinal twisting) - Shashankasana   (rabbit pose)   - Bhujangasana (serpent pose) - Shalbhasana   (locust pose)   - Halasana (plough pose) | **Pranayama - 20**  **mins**   - Sequential Nadisodhan pranayama (alternate nostril breathing) – 10 mins - 3 rounds - Bhastrika pranayama (bellows breath)-5 mins - 2 rounds - Bhramari pranayama (humming bee breath) - 5 mins - 10 rounds | **Dhyana and**  **relaxation**  **practices - 25 mins**   - Soham meditation - 8 mins followed by - OM meditation - 2 mins - 10 rounds - Instant relaxation with   Gayatri mantra - 5 mins   - Savasana (corpse pose) – 10 mins | I = 85 mins daily X 7 weeks (excluding Sundays) (~40 days) | -Centre-based group sessions delivered and supervised by a qualified yoga teacher. | Cleansing techniques:   - Kunjal kriya (stomach cleansing) - twice/week - Jal Neti (nasal cleansing) - thrice/week   -Queries and feedback - 5 mins |
| Ward, 2018^57^ | NR | 8 weeks | **Asana - 38 mins**   - Pawanmuktasana 1 (wind release pose) - 10 mins - Supine, seated and standing postures - 28 mins | **Pranayama - 7 mins**   - Centring and breathing practice - 7 mins | **Dhayana and relaxation practices - 15 mins**   - Guided relaxation - 15 mins | I = 60 mins/week X 8 weeks | -Centre-based group sessions delivered and supervised by a qualified yoga instructor and class assistant and home-based individual sessions.  -Thrice/week home-based yoga practice was encouraged.  -Use of props (foam, block, belt etc).  -Home-based practice guided via CD. | - Check in - 5 mins - Introduction to the class lesson and yoga philosophy theme - 5 mins - Warm-up practice - Closing discussion - 5 mins |
| Gautam, 2019,2020,2021,2022,2023^58-62^ | Based on Patanjali’s Ashtanga yoga for joint inflammation | 8 weeks | **Asana - 20 mins**  Standing poses - 5 mins   - Trikonasana (triangle pose) - Katichakrasana (waist rotating pose) - Tadasana (mountain pose) - Virabhadrasana (warrior pose)   Sitting poses - 5 mins   - Gomukhasana (cow face pose) - Paschimottanasana (seated forward bend) - Shashankasana (hare pose) - Vakrasana (spinal twist pose)   Prone poses - 5 mins   - Ek-pada-shalabhasana (locust pose) - Bhujangasana (cobra pose) - Poorna shalabhasana (full locust pose) - Makarasana (crocodile pose)   Supine poses- 5 mins   - Uttanapadasana (intense stretch of legs) - Setu bandhasana (bridge pose) - Pavanamuktasana (wind-relieving pose) - Matsyasana (fish pose) | **Pranayama - 20 mins**   - Kapalbhati kriya (skull-shining breath) - Ujjayi breath (diaphragmatic breath) - Nadi shodhana (alternate nostril breathing) - Bhramari (bee breath) | **Dhayana and relaxation**  **practices - 28 mins**   - Dhyana (meditation) - 15 mins - Savasana (corpse pose) - 10 mins - Nada anusandhana, OM meditation - 3 mins | I = 68 mins X 5 times/week X 8 weeks | -Centre-based group sessions delivered and supervised by  well-qualified yoga instructors.  -Adherence was monitored with the participant's diary and yoga teacher’s remarks at each visit. | -Session preparation  instructions - 2 mins  -Starting prayer - 5  mins  -Yogic sukshma  vyama + yogic sthool  vyama – 10 mins  -Interactive  session/self-  directed learning - 30  mins  -Interactive session/self-directed learning - 30 mins  -Shanti mantra  (closing  prayer) - 5 mins  -Encouragement for long term yoga. |
| Ganesan, 2020^63^ | NR | 12 weeks | **Asana - 8 mins**   - Tadasana (mountain pose) - Katichakrasana (lateral arc pose) - Konasana (angle pose) - Urdhwa hastottanasana (upstretched arms posture) - Pavanamuktasana (wind removing pose) - Bhujangasana (cobra pose) | **Pranayama – 6 mins**   - Nadishodhana pranayama   (alternate nostril breathing)   - Chandrabhedi pranayama (left   nostril breathing)   - Bhramari (humming bee breathing) | **Dhyana and**  **relaxation**  **practices - 5 mins**   - Dhyana (OM meditation) - Savasana (corpse pose, palms up) | I = 19 mins X 3 times/week X 12 weeks | -Centre-based group sessions delivered and supervised by qualified and experienced yoga instructors and home-based individual sessions.  -Home-based practice was encouraged and monitored by phone calls. | -Warm up - 2 mins  -Sukshma vyama – 9 mins |
| Pukšić, 2021^64^ | Based on the Yoga in Daily Life system (Level 1 -Sarvahita Asanas) was developed by Indian yoga teacher Vishwaguru Paramhans Sri Swami Maheshwaranda. | 12 weeks | **Asana - NR** | **Pranayama - 70 mins**   - Breathing   exercises (included  abdominal breathing  and consecutively  complete yogic  breath (utilising  abdominal, thoracic  and clavicular part of  the breath)) - 50–60  mins   - Nadi   shodhana pranayama  (alternate nostril  breathing - 10 mins | **Dhyana and relaxation**  **practices - 25 mins**   - Guided   relaxation- in a  supine position  Anantasana (sleeping Vishnu pose) - 5–10 mins   - Short   relaxation - 5 mins   - Self-   inquiry meditation – 5-10 mins   - Closing   OM meditation | I = 95 mins X 2 times/week X 12 weeks | -Centre-based group sessions delivered and supervised by a  rheumatologist  and qualified  yoga instructor.  -Pranayama  and meditation  were performed  sitting on exercise  balls.  -Home practice advised but not formally monitored. | -Postures adapted for functional limitations |

NR: Not reported (Only the terms- Asana, Pranayama or Dhyana and relaxation practices were mentioned but not no details provided)

PR: Pulse rate, BP: Blood pressure, LC: Lymphocyte count, UA: Serum uric acid, AIIMS: All India Institute of Medical Sciences, ACYTER: The Advanced Centre for Yoga Therapy Education

and Research, JIPMER: Jawaharlal Institute of Postgraduate Medical Education and Research

Table S2: Methodological assessment of the included studies.

| **Author and year** | **Q1** | **Q2** | **Q3** | **Q4** | **Q5** | **Q6** | **Q7** | **Q8** | **Q9** | **Q10** | **Q11** | **Q12** | **Q13** | **Total % of “Y” (Excluding “N/A”)** |
| --- | --- | --- | --- | --- | --- | --- | --- | --- | --- | --- | --- | --- | --- | --- |
| Singh, 2011^56^ | U | U | Y | U | N/A | U | U | Y | N | N | U | U | Y | 25 |
| Ward, 2018^57^ | Y | Y | N | U | N/A | Y | Y | Y | U | Y | U | U | Y | 58 |
| Gautam, 2019, 2020, 2021, 2022, 2023^58-62^ | Y | Y | N | N | N/A | Y | Y | Y | U | Y | U | Y | Y | 66 |
| Ganesan, 2020^63^ | Y | U | Y | U | N/A | U | U | U | U | Y | U | Y | Y | 42 |
| Pukšić, 2021^64^ | Y | U | Y | U | N/A | Y | Y | U | U | Y | U | Y | Y | 58 |

This tool uses a series of criteria that can be scored as being met (yes), not met (no), unclear or not applicable (n/a).

Y=yes; N=no; U=unclear; NA=not applicable.

In real practice, it is practically impossible to blind the yoga provider. So, the response to question 5 of the checklist was marked as N/A (not applicable) in our methodological assessment.

JBI critical appraisal checklist for randomised controlled trials: Q1. Was true randomisation used for assignment of participants to treatment groups? Q2. Was allocation to treatment groups concealed? Q3. Were treatment groups similar at baseline? Q4. Were participants blind to treatment assignment? Q5. Were those delivering treatment blind to treatment assignment? Q6. Were treatment groups treated identically other than the intervention of interest? Q7. Were outcomes assessors blind to treatment assignment? Q8. Were outcomes measured in the same way for treatment groups? Q9. Were outcomes measured in a reliable way? Q10. Was follow-up complete and if not, were differences between groups in terms of their follow up adequately described and analysed? Q11. Were participants analysed in the groups to which they were randomised? Q12. Was appropriate statistical analysis used? Q13. Was the trial design appropriate, and any deviations from the standard RCT design (individual randomisation, parallel groups) accounted for in the conduct and analysis of the trial?

Table S3: Extracted outcome data.

| **Author and year** | **Pain (Post-intervention)**  **Mean ± SD** | **Function (Post-intervention)**  **Mean ± SD** | **Disease activity (Post-intervention)**  **Mean ± SD** | **Adverse events related to intervention (including serious adverse events)** | **Measurement time point at the end/closest to the end of intervention for disease activity score and/or pain and/or function** |
| --- | --- | --- | --- | --- | --- |
| Singh, 2011^56^ | *SDPIS (0-5)*  *I=0.20±0.52*  *C=1.92± 0.76* | - | - | NR | 40 days or approximately 7 weeks |
| Ward, 2018^57^ | *VAS (0-100 mm)*  *I=33±21.00*  *C=33±32.00* | *HAQ-DI (0-3)*  *I=0.35±0.35*  *C= 0.83±0.76* | *CDAI*  *I=11.5±7.30*  *C=9.6±7.60* | Nausea: I=6 (6 events reported by 1 participant)  -No serious AEs | 9 weeks |
| Gautam, 2019^58^ | **-** | *HAQ-DI*  *I=0.44±0.36*  *C=0.50±0.38* | DAS28-ESR  I=4.42±0.93  C=4.8±0.87 | NR | 8 weeks |
| Gautam, 2020^59^ | **-** | **-** | DAS28-ESR  I=4.5±0.90  C=4.9±0.80 | NR | 8 weeks |
| Gautam, 2021^60^ | **-** | HAQ-DI  I=0.45±0.30  C=0.52±0.40 | DAS28-ESR  I=4.5±0.90  C=4.8±0.85 | NR | 8 weeks |
| Gautam, 2022^61^ | **-** | **-** | *DAS28-ESR*  *I=4.1±0.90*  *C=4.5±1.30* | NR | 8 weeks |
| Gautam, 2023^62^ | **-** | **-** | DAS28-ESR  I=3.8±0.90  C=4.3±1.30 | NR | 8 weeks |
| Ganesan, 2020^63^ | **-** | **-** | *DAS28*  *I=2.99±0.78*  *C=3.49±0.78* | NR | 12 weeks |
| Pukšić, 2021^64^ | *VAS (0-10 cm)*  *I=2.42±1.79*  *C=3.63±2.57*  SF-36 (0-100)  Bodily pain  I=69.48**±**19.48  C=54.48±19.04 | *SF-36 (0-100)*  *I=70.83±19.82*  *C=59.36±20.50* | *DAS28CRP (used interchangeably with DAS28ESR)*  *I=2.24±0.59*  *C=2.82±0.99* | I=1(Persistent positional vertigo (supine position)  C=none  -Serious AEs-none | 12 weeks |

SDPIS: Simple Descriptive Pain Intensity Scale, CRP: C-reactive protein, VAS: Visual Analogue Scale, HAQ-DI: Health Assessment Questionnaire Disability Index, CDAI: Clinical Disease Activity Index, DAS-Disease activity score, ESR: Erythrocyte sedimentation rate, IL: Interleukin, TNF- Tumour necrosis factor, HLA-G: Human leukocyte antigen G, SF-36: Short Form 36, NR: Not reported

Data used for meta-analysis have been represented in Italics.

Table S4: Description of outcome measurement scales, including scoring, and interpretation.

| **Category** | **Scale** | **Description** | **Scoring and interpretation** |
| --- | --- | --- | --- |
| **Disease activity**  **Score** | Clinical Disease Activity Index  (CDAI)^1^ | - To measure disease activity. - Consists of the number of tender joints (0–28) (clinician-reported), number of swollen joints (0–28) (clinician-reported), patient global disease activity (self-reported), and physician global assessment of the disease activity (clinician-reported). | - Score ranges from 0 to 76. - Remission: ≤2.8 - Low disease activity: >2.8 to ≤10 - Moderate disease activity: >10 to ≤22 - High disease activity: >22.1 |
|  | Disease Activity Score in 28 joints  (DAS28) [DAS28 - Erythrocyte  sedimentation rate (ESR) and  DAS28 - C-reactive protein  (CRP)]^2,3^ | - To measure disease activity. - Consists of the number of tender joints (0–28) (clinician-reported), number of swollen joints (0–28) (clinician-reported), patient global health on a Visual Analogue Scale (VAS) of 0–10 (self-reported), and measurement of acute phase reactants (either ESR or CRP) (lab-based). - DAS28-CRP is a modification of DAS28-ESR, with CRP responding more rapidly to inflammation changes. | - Score ranges from 0 to 9.4. - Remission: <2.6 - Low disease activity: ≥2.6 to ≤3.2 - Moderate disease activity: >3.2 to ≤5.1 - High disease activity: >5.1 |
| **Pain** | Simple Descriptive Pain Intensity  Scale (SDPIS)^4^ | - To measure pain intensity. - Self-reported. | - Score ranges from 0 (no pain) to 5 (worst possible pain). |
|  | Visual Analogue Scale (VAS)^5^ | - To measure pain intensity. - A 10 cm ruler is used to mark pain intensity. - Self-reported. | - Score ranges from 0 (no pain) to 100 (worst possible pain). - Score is determined by measuring the distance (in millimetres or centimetres) from the zero point to the patient's mark on the scale. |
| **Function** | Health Assessment Questionnaire  Disability Index (HAQ-DI)^6^ | - To measure functional ability in activities of daily living. - Comprises 20 items across 8 domains, including dressing, rising, eating, walking, hygiene, reach, grip, and usual activities. - Self-reported. | - Each domain has a set of questions scored from 0 (no disability) to 3 (unable to perform activity). - Overall score is the mean of the 8 domain scores. |
|  | Short Form-36 Health Survey (SF-  36)^7^ | - To measure overall health status. - Consists of 36 items across 8 domains, including vitality, physical functioning, bodily pain, general health, physical role functioning, emotional role functioning, social role functioning, and mental health. - Self-reported. | - Each domain is scored separately from 0 (worst possible health) to 100 (best possible health). - Scores are weighted sums of items in each domain. |

1. Aletaha D, Smolen J. The Simplified Disease Activity Index (SDAI) and the Clinical Disease Activity Index (CDAI). *Rheumatology* (Oxford). 2005;44(5):592-8.

2. Prevoo ML, van 't Hof MA, Kuper HH, van Leeuwen MA, van de Putte LB, van Riel PL. Modified disease activity scores that include twenty-eight-joint counts. *Arthritis Rheum*. 1995;38(1):44-

48.

3. Inoue E, Yamanaka H, Hara M, Tomatsu T, Kamatani N. Comparison of Disease Activity Score (DAS)28-erythrocyte sedimentation rate and DAS28-C-reactive protein threshold values. *Ann*

*Rheum Dis*. 2007;66(3):407-9.

4. Riegel B. Pain Assessment: Simple Descriptive Pain Intensity Scale. Burn Survivors Throughout The World, Inc. Available from: <https://www.burnsurvivorsttw.org/articles/painass2.html>

5. Hawker GA, Mian S, Kendzerska T, French M. Measures of adult pain: Visual Analog Scale for Pain (VAS Pain), Numeric Rating Scale for Pain (NRS Pain), McGill Pain Questionnaire

(MPQ), Short-Form McGill Pain Questionnaire (SF-MPQ), Chronic Pain Grade Scale (CPGS), Short Form-36 Bodily Pain Scale (SF-36 BPS), and Measure of Intermittent and Constant

Osteoarthritis Pain (ICOAP). *Arthritis Care Res (Hoboken)*. 2011;63(Suppl 11):S240-S252.

6. Bruce B, Fries JF. The Health Assessment Questionnaire (HAQ). Clin Exp Rheumatol. 2005;23(5):S14-8.

7. Ware JE Jr, Sherbourne CD. The MOS 36-item short-form health survey (SF-36): I. Conceptual framework and item selection. *Med Care*. 1992;30(6):473-83.

| Table S5: Summary of findings. | | | | | | |
| --- | --- | --- | --- | --- | --- | --- |
| **Yoga in addition to standard medical treatment for RA** | | | | | | |
| **Patient or population:** People with RA  **Setting:** Primary, secondary, tertiary or community  **Intervention:** Yoga (in addition to standard medical treatment for RA)  **Comparison:** Non-yoga comparator (i.e., wait-listed control or health education on RA) | | | | | | |
| Outcomes | **Anticipated absolute effects^*^** (95% CI) | | Relative effect (95% CI) | № of participants (studies) | Certainty of the evidence (GRADE) | Comments |
|  | Risk with comparator | Risk with yoga (in addition to standard medical treatment) |  |  |  |  |
| Pain | - | SMD 1.06 lower (2.6 lower to 0.48 higher) | - | 163 (3 RCTs) | ⨁◯◯◯  Very low^a,b,^ |  |
| Function | - | SMD 0.42 lower (0.76 lower to 0.07 lower) | - | 155 (3 RCTs) | ⨁⨁⨁◯  Moderate^b^ |  |
| Disease activity | - | SMD 0.45 lower (0.75 lower to 0.15 lower) | - | 389 (4 RCTs) | ⨁⨁⨁◯  Moderate^b^ |  |
| ***The risk in the intervention group** (and its 95% confidence interval) is based on the assumed risk in the comparison group and the **relative effect** of the intervention (and its  95% CI). **CI:** confidence interval; **SMD:** standardised mean difference | | | | | | |

**GRADE Working Group grades of evidence**

**High certainty:** we are very confident that the true effect lies close to that of the estimate of the effect.

**Moderate certainty:** we are moderately confident in the effect estimate: the true effect is likely to be close to the estimate of the effect, but there is a possibility that it is substantially

different.

**Low certainty:** our confidence in the effect estimate is limited: the true effect may be substantially different from the estimate of the effect.

**Very low certainty:** we have very little confidence in the effect estimate: the true effect is likely to be substantially different from the estimate of effect.

Explanations a. Statistical heterogeneity, b. Small sample size

Table S6: Yoga practices used in effective interventions for disease activity score, pain, and/or function.

| **Asana** | | | | **Pranayama** | | | **Dhyana and relaxation practice** | | |
| --- | --- | --- | --- | --- | --- | --- | --- | --- | --- |
| **Sanskrit**  **Name** | **English**  **Name** | **Type of yoga**  **(standing,**  **sitting,**  **supine or**  **prone)** | **Effective studies**  **reporting use of the**  **asana for disease**  **activity score**  **and/or pain and/or**  **function** | **Sanskrit**  **Name** | **English**  **name** | **Effective studies**  **reporting use of the**  **pranayama for**  **disease activity**  **score and/or**  **pain and/or**  **function** | **Sanskrit**  **name** | **English**  **Name** | **Effective studies**  **reporting use of the**  **dhyana and**  **relaxation**  **practice for disease**  **activity score and/or**  **pain and/or function** |
| Pawanmuktasana | Wind  relieving  pose | Supine pose | Disease activity score^58-62,63^  Pain^56^  Function^57^ | Nadishodhana  pranayama | Alternate  nostril  breathing | Disease activity  score^58-62,63,64^  Pain^56^  Function^64^ | Nadanusandhana | OM meditation/ A,U,M and A-U-M Kara chanting | Disease activity  score^58-62,63,64^  Pain^56^  Function^64^ |
| Marjari asana | Cat pose | Prone pose | Pain^56^ | Chandrabhedi  pranayama | Left nostril  breathing | Disease activity score^63^ | Savasana | Corpse  pose,  palms up | Disease activity  score^58-62,63^  Pain^56^ |
| Vakranasana | Twisted  pose | Seated pose | Disease activity score^58-62^  Pain^56^ | Bhramari  Pranayama | Humming bee breathing | Disease activity  score^58-62,63^  Pain^56^ | Anantasana | Sleeping Vishnu pose/ side- reclining leg lift | Disease activity score^64^  Function^64^ |
| Shashankasana | Rabbit pose  or hare pose | Seated pose | Disease activity score^58-62^  Pain^56^ | Dirga/Mahat  pranayama | Complete yogic breath  (utilising abdominal,  thoracic and clavicular  part of the breath) | Disease activity score^64^  Function^64^ | NR | Short  relaxation | Disease activity score^64^  Function^64^ |
| Bhujangasana | Cobra pose | Prone pose | Disease activity score^58-62,63^  Pain^56^ | Kapalbhati  Pranayama | Skull shining  breathing | Disease activity  score^58-62^ | NR | Self-inquiry  meditation | Disease activity score^64^  Function^64^ |
| Poorna  shalabhasana | Locust pose | Prone pose | Disease activity score^58-62^ | Ujjayi  Pranayama | Victorious  breath | Disease activity score^58-62^ | Soham mantra | Soham meditation | Pain^56^ |
| Halasana | Plough pose | Supine pose | Pain^56^ | Bhastrika  Pranayama | Bellows  breathing | Pain^56^ | NR | Instant  relaxation  with Gayatri  mantra | Pain^56^ |
| Konasana | Angle pose | Standing pose | Disease activity score^63^ | NR | Centring and  breathing  practice | Function^57^ | NR | Guided  relaxation | Function^57^ |
| Urdhwa  Hastottanasana | Upstretched  arms  posture | Standing pose | Disease activity score^63^ |  |  |  |  |  |  |
| Trikonasana | Triangle  pose | Standing pose | Disease activity score^58-62^ |  |  |  |  |  |  |
| Katichakrasana | Lateral arc pose | Standing pose | Disease activity score^58-62,63^ |  |  |  |  |  |  |
| Tadasana | Mountain pose | Standing pose | Disease activity score^58-62,63^ |  |  |  |  |  |  |
| Virabhadrasana | Warrior  pose | Standing pose | Disease activity score^58-62^ |  |  |  |  |  |  |
| Gomukhasana | Cow face  Pose | Seated pose | Disease activity score^58-62^ |  |  |  |  |  |  |
| Paschimottanasana | Seated  forward  bend pose | Seated pose | Disease activity score^58-62^ |  |  |  |  |  |  |
| Ek-pada-  shalabhasana | Locust pose | Prone pose | Disease activity score^58-62^ |  |  |  |  |  |  |
| Makarasana | Crocodile  Pose | Prone pose | Disease activity score^58-62^ |  |  |  |  |  |  |
| Uttanapadasana | Intense  stretch  of legs pose | Supine pose | Disease activity score^58-62^ |  |  |  |  |  |  |
| Setu bandhasana | Bridge pose | Supine pose | Disease activity score^58-62^ |  |  |  |  |  |  |
| Matsyasana | Fish pose | Supine pose | Disease activity score^58-62^ |  |  |  |  |  |  |

NR: Not reported **Standing pose:** yoga poses practiced with one or both feet on the ground, and the body more or less upright, **Sitting pose:** Yoga poses practiced in seated position, **Supine**

**pose:** Yoga poses practiced in sleeping position, **Prone pose:** Yoga poses performed with the belly or torso touching or facing the floor.
